# 1-Methyl-4-Phenylpyridinium Stereotactic Infusion Completely and Specifically Ablated the Nigrostriatal Dopaminergic Pathway in Rhesus Macaque

**DOI:** 10.1371/journal.pone.0127953

**Published:** 2015-05-26

**Authors:** Xiaoguang Lei, Hao Li, Baihui Huang, Joshua Rizak, Ling Li, Liqi Xu, Li Liu, Jing Wu, Longbao Lü, Zhengbo Wang, Yingzhou Hu, Weidong Le, Xingli Deng, Jiali Li, Yonggang Yao, Lin Xu, Xintian Hu, Baorong Zhang

**Affiliations:** 1 Department of Neurology, Second Affiliated Hospital, School of Medicine, Zhejiang University, Hangzhou, Zhejiang, China; 2 Key Laboratory of Animal Models and Human Disease Mechanisms of the Chinese Academy of Sciences & Yunnan Province, Kunming Institute of Zoology, Chinese Academy of Sciences, Kunming, Yunnan, China; 3 University of the Chinese Academy of Sciences, Beijing, China; 4 Medical Imaging Department, Kunming General Hospital of PLA, Kunming, Yunnan, China; 5 Kunming Primate Research Center, Kunming Institute of Zoology, Chinese Academy of Sciences, Kunming, Yunnan, China; 6 Institute of Health Sciences, Shanghai Institutes for Biological Sciences, Chinese Academy of Sciences, Shanghai, China; 7 Neurosurgery Department, 1st Affiliated Hospital of Kunming Medical University, Kunming, Yunnan, China; 8 CAS Center for Excellence in Brain Science, Chinese Academy of Sciences, Shanghai, China; Emory University, UNITED STATES

## Abstract

**Introduction:**

Complete and specific ablation of a single dopaminergic (DA) pathway is a critical step to distinguish the roles of DA pathways in vivo. However, this kind of technique has not been reported in non-human primates. This study aimed to establish a lesioning method with a complete and specific ablation.

**Method:**

A carefully designed infusion route based on a MRI stereotactic technique was developed to deliver the highly selective dopaminergic toxin 1-methyl-4-phenylpyridinium (MPP^+^) unilaterally into multiple sites of compact part of substantia nigra (SNc) and striatum in monkeys. The nigrostriatal DA pathway was selected because lesioning of this pathway may induce symptoms that are suitable for evaluation. The pathological, behavioral, neuropharmacological, and clinical laboratorial data were collected to evaluate the lesioning effects.

**Result:**

Pathological examination revealed a complete ablation of tyrosine hydroxylase positive (TH+) neurons in the SNc, while preserving intact TH+ neurons in the ventral tegmental area (VTA) nearby. TH+ projections in the striatum were also unilaterally lost. The monkeys displayed stable (>28 weeks) rotations and symptoms which were expected with loss of DA neurons in the SNc, with rest tremor being an exception. No item implied the presence of a severe side effect caused by the operation or the intracerebral MPP^+^ infusion. The results suggested that rest tremor may not directly rely on the nigrostriatal pathway.

**Conclusion:**

Taken together, in addition to providing a specific nigrostriatal DA lesioned model, this method, combined with brain stimulation or other techniques, can be applied as a powerful tool for the complete lesion of any desired DA pathway in order to study its specific functions in the brain.

## Introduction

Dopamine (DA) is a key neurotransmitter in the body. In the central nervous system (CNS), dopaminergic neurons form distinct DA systems which play important roles in movement, emotion, reward behavior, sleep, memory, endocrine function, and sensory processing [1]. For decades, researchers have tried several methods to lesion DA pathways to investigate the functions of dopamine and to understand the pathogenesis of Parkinson’s disease (PD) [2]. However, the exact roles of different DA pathways in normal or PD states have not been fully understood, despite the pathophysiological basis of the disease being associated to nigrostriatal DA neuron loss [3]. This is notably due to the lack of accurate methods to lesion a specific DA pathway. For example, the monkey model induced by injecting 1-methyl-4-phenyl-1,2,3,6-tetrahydropyridine (MPTP) via muscle or the carotid artery [4], targets most DA pathways and non-specifically lesions DA tissue, leading to a number of side effects [4–6]. This widespread damage fundamentally challenges the ability to correlate effects to specific dopamine pathways, thereby limiting the application of future research.

**Fig 1:**
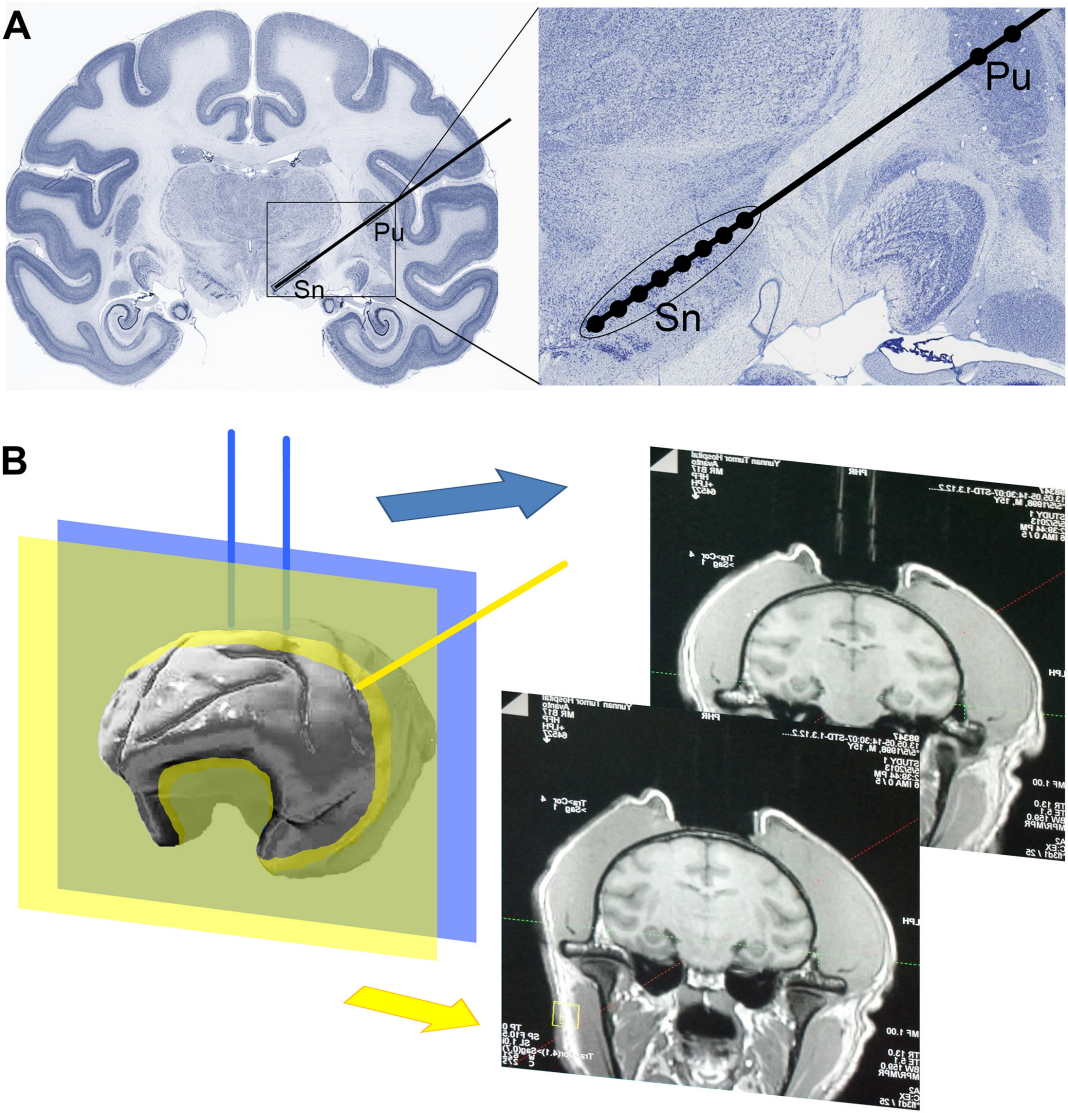
Route of MPP^+^ infusion. **(A)** Designed route of MPP^**+**^ infusion in a coronal section of the rhesus monkey brain [47]. The route (black line) can penetrate both SNc and striatum (particularly the putamen, Pu). Black dots in the right panel indicate the ten sites of MPP^**+**^ infusion. **(B)** Glycerol tubes (blue lines) detectable in MRI serve as markers. The final plane (yellow plane) of the infusion route (yellow line) is determined based on the distance between the coronal plane of targeted SNc and the coronal plane (blue plane) of glycerol marker (blue lines).

This study set out to develop a new method to provide complete and specific lesions of individual DA pathways for the study of specific functions of each individual DA pathway in the brain. It targeted the complete lesion of the nigrostriatal DA pathway, while leaving other DA pathways intact, as this lesion will help to accurately understand the specific role of the nigrostriatal pathway, to develop specific models and to design new clinical approaches for disease treatment. This method can be easily combined with deep brain stimulation, electrophysiological recording, cell transplantation and other experimental techniques. In this approach, a new infusion route was designed (Fig 1A) to deliver the neurotoxin for an accurate lesion of the SNc. A Magnetic Resonance Imaging (MRI) based stereotactic method (Fig 1B) was optimized to improve the accuracy of stereotaxis. 1-methyl-4-phenylpyridinium (MPP^+^), the toxic metabolic product of the classic neurotoxin MPTP, was utilized to guarantee the direct and specific lesioning effect in rhesus macaque (*macaca mulatta*).

## Methods

### Ethics and Animals

This experiment was performed in accordance with the Guide for the Care and Use of Laboratory Animals [7] and the ARRIVE Guidelines for Reporting Animal Research [8]. The experiment and all related protocols were approved by the Ethics Committee of the Kunming Institute of Zoology (AAALAC accredited), Chinese Academy of Sciences. All efforts were made to minimize suffering.

Monkeys (rhesus macaque, *macaca mulatta*) were pre-screened and purchased from Kunming Biomed International (AAALAC accredited, Kunming, China). Monkeys who were disabled, unable to cooperate, unhealthy, poor in movement, displayed stereotypical movements or bizarre behavior were excluded. Six male macaques (macaca mulatta) 12–15 year-old (14.0±1.1), weighing 7-16kg (9.9±3.2) were included in this study (Table 1).

**Table 1:** Detailed Monkey Information and Study Parameters.

ID	Gender	Age (year)	Weight (kg)	Group
**#1**	Male	12	15.84	MPP^+^
**#5**	Male	15	9.49	Saline
**#6**	Male	15	10.95	MPP^+^
**#9**	Male	14	7.05	MPP^+^
**#10**	Male	14	7.98	MPP^+^
**#14**	Male	14	7.88	Saline

Each monkey had its own cage (80×80×80 cm) in a big room controlled by a 12/12-hour day-night lighting system (lights on at 7:00 A.M., light intensity of 100–200 lux) and a McQuay air conditioning system (temperature of 18–26°C, humidity of 30% -70%). The monkeys could communicate with others. Food including grains, vegetables and fruits was provided at fixed times daily, while fresh water was available *ad libitum*. Food waste and excrement under the cages were washed daily. There was a step and a toy in each cage to provide environment enrichment. The toys (such as a plastic hollow ball and a plastic bone for child use) were changed every week. A full-time attending veterinarian monitored the monkey’s health.

The pre-estimation of statistical power and sample size was done in NCSS PASS (free trial version) for measuring the monkeys’ Kurlan’s score. A repeated measures design was used with one between group factor and one within group factor (time) for two groups. This design achieved ~100% power to test the group factor if a Geisser-Greenhouse Corrected F Test is used, achieved 95% power to test the time factor if a Geisser-Greenhouse Corrected F Test is used, and achieved 97% power to test the group×time interaction if a Geisser-Greenhouse Corrected F Test is used.

After baseline data were collected, monkeys were randomly divided into an MPP^+^ group (N = 4) and a saline sham-operation control group (N = 2). MPP^+^ (MPP^+^ iodide, D048, Sigma, USA) dissolved in saline was used for administration in the MPP^+^ group, while saline (Anting, Shanghai, China) alone was administered to the control group. Iodide has been shown to have no additional lesioning effect to DA neurons [9].

### Monkey Stereotaxis

#### Glycerol marker for MRI

Glycerol tubes are detectable in an MRI and therefore served as markers when attached to the monkey’s skull (Fig 1B, blue lines). The rigid glass tubes (4 cm long, internal diameter 0.5 mm, external diameter 0.9 mm) were filled with glycerol and then sealed with an alcohol burner. The monkey was anesthetized with hydrochloric acidulated ketamine (0.3 g/2 ml, 0.5–0.7 ml, i.m., Jinlu, Shenyang, China) 10 minutes after an atropine sulfate injection (0.5 mg/1 ml, 1 ml, i.m., Runhong, Xinzhen, China), and maintained with pentobarbital (40 mg/ ml, 20 mg/ kg, i.m., Pertobarbital Sodium, Fluka, Germany). After the eyelash reflex had disappeared, the monkey was transferred into an animal operation room, which had been disinfected with ultraviolet (UV) light for no less than 30 minutes prior to the surgery. Fur from the operating field was then clipped off to prevent contamination and the monkey’s heads was fixed on a stereotaxis apparatus (Model 1504 Heavy-Duty Research Stereotaxic for Monkeys, KOPF, USA). The skin was sterilized with iodophor and 75% alcohol. Strictly following an aseptic technique, an incision was made on scalp. Tissue under the incision was cleaned and the surface of the skull was scrubbed with saline and 3% hydrogen peroxide.

A three-dimensional rectangular coordinate system was established using the X axis (intersection of sagittal and horizontal planes), Y axis (intersection of coronal and horizontal planes) and Z axis (intersection of sagittal and coronal planes). The glass tube markers were anchored at coordinates obtained from the Rhesus monkey brain atlas [10]. For monkeys #9, #10 and #14, one glass tube was fixed on the skull at the extension line of the theoretical infusion route [located on the X axis: 0.93cm before A/P zero; Y axis: 3.11 cm left to sagittal plane; Z axis: equal to the height of skull top at the center front point (x = 0.93 cm before A/P, y = 0 to sagittal plane); oblique angle to horizontal plane: 54°]; for monkeys #1, #5 and #6, two glass tubes, 1 cm apart from each other (Fig 1B, blue lines), were fixed vertically on the coronal plane (Fig 1B, blue plane) of the theoretical infusion route (X axis: 0.93 cm before A/P zero; Y axis: distance of 1 cm to each other, and 0.5 cm to the sagittal plane; Z axis: skull top, oblique angle to horizontal plane: 54°). Although the two approaches utilized different locations to anchor the marker tubes, both methods provided sufficient information to calibrate the final coordinates because their mathematical bases were the same. The approach utilizing two-tubes was, in retrospect, more convenient but required more calculation to determine the angles of infusion than the one-tube approach.

Shallow holes were made in the skull with an electric dental drill to hold the base of the glass tubes. An electrode manipulator (Electrode Manipulators Models 1460, 1460–61, KOPF, USA) was used to hold the tubes. Dental cement was then poured to fix the tubes to the skull. Penicillin (1600KU, i.m., Harbin Pharmaceutical Group Sixth Pharmaceutical Factory, Harbin, China) was administered for at least three consecutive days after the marker anchoring and the infusion operations to prevent infection.

#### MRI and coordinate adjustment

One day (for monkeys #1, #5 and #6) or two days (for monkeys #9, #10 and #14) after the glycerol marker operation, the monkeys were anesthetized again and their brains scanned in an MRI [Siemens Avanto 1.5T, with a 3D FLASH (Fast Low Angle Shot) Spoiled Gradient Echo Sequence under the following parameters: slice 1.0 mm; distance factor 0.2mm/20%; FOV 150×122 mm, resolution 320×320; TR 13.0 ms; TE 5.07 ms; averages 3]. The coronal plane containing glass tubes (Fig 1B, yellow plane) in the three-dimensional reconstruction of the MRI were used as the reference to build the three-dimensional rectangular coordinate system. The medial part of substantia nigra (SN) in the mesencephalon, which had lower signal intensity in comparison to the surrounding area, was set as the initial site of infusion.

For monkeys #9, #10 or #14, the final plane of the infusion route was determined based on the distance between the coronal plane of targeted SNc and the coronal plane of glycerol marker. The height adjustment was based on the distance between the extension line of the glycerol tube and initial infusion site. The oblique angle of the final route was determined based on the position of the targeted striatum. The length and depth of the infusions in the SNc and striatum were measured along the final route.

For monkeys #1, #5 or #6, the final plane of the infusion route (Fig 1B, yellow plane) was determined based on the distance between the initial infusion site and the coronal plane of glycerol marker (Fig 1B, blue plane), the distance between the initial infusion site and the skull directly above, and the distance between the initial infusion site and the median sagittal plane. The oblique angle of the final route (Fig 1B, yellow line) was determined based on the position of targeted striatum. The length and depth of the infusions in SNc and striatum were measured along the final route.

#### Stereotactic infusion of MPP^+^

One day (#1, #5 and #6) or two days (#9, #10 and #14) after MRI scanning, monkeys were given infusions of either MPP^+^ or saline, as a control, via the routes identified above. The animals were anesthetized following the standard procedure mentioned above and the dental cement and glycerol tubes were removed from the skull. After cleaning the skull with 3% hydrogen peroxide and saline, a hole (d = 1 mm) was made by dental drill through the skull based on the adjusted coordinates determined from the MRI scan. A microsyringe (10 μl, Anting, Shanghai, China) filled with either MPP^+^ solution or saline was fixed on the electrode manipulator (Electrode Manipulators Models 1460, 1460–61, KOPF, USA) and was slowly inserted (< 1 cm/min) down the infusion route and into the initial infusion site by a pusher. The microsyringe was maintained for 1 min in the infusion site (SNc) before each infusion began. Then the MPP^+^ group or saline group were separately administered either MPP^+^ (100 mg/ml, 10 μl) or saline (10 μl) at a rate of < 1 μl /min. A 1 μl volume of each agent was administered at each of eight infusion sites in the SNc at the same interval (between 0.06 to 0.08 cm, individualized for each monkey) and at two infusion sites in the striatum. Two minutes after each infusion, the microsyringe was retracted (<1 cm/min) to the next adjacent site. After all infusions to a monkey were completed, the microsyringe was pulled out (<1 cm/min) and the incision was sutured and cleaned. Then antibiotics were administered as described above.

### Data collection

#### Kurlan’s Score

The rating was processed according to the accepted standard [11]. Videos of home-caged behavior without interference were captured by high-resolution cameras (HDR-XR260, SONY). One hour videos were captured daily for the first three days before and after the infusion operation, then they were captured once every week for five months and then once a month in subsequent months. The recording and video collection was performed by technicians who were blind to the grouping. The modified Kurlan’s scale (Part A; measuring Parkinsonian features with a total score of 20 where a higher score indicates more severe Parkinsonism) [12], which has been widely accepted as a valued scale for PD research in the old world monkeys [13], was used for the evaluation of Parkinsonism severity in the monkeys. The scoring was based on the whole 1 hour video of each recording. The scorer was previously trained and was specialized in Kurlan’s scoring. The scorer was also blind to the animal’s grouping and the capturing time of each video.

#### Spontaneous rotation and APO-induced rotation

For spontaneous rotation, the rotations were counted in 30-minute sections (15–45 minute after recording began) of each video recording mentioned above. For APO-induced rotation, a new series of video were captured after the monkeys were administrated with APO [R-(-)-Apomorphine hydro-chloride hemihydrate, A4393-100MG, Sigma, USA]. APO administrations were performed once a week from week 1–8 and then every month after the infusion operation. APO (2 mg) was dissolved in a Vitamin C solution (0.5 mg / 2 ml) 5 minutes before being infused in to the monkey (1 mg/ml; 2 ml, i.m.). The videos were captured 15 minutes after the APO injection and were collected for 1 hour each. APO-induced rotations were also counted in 30-minute sections (15–45 minute after recording began) in the videos. If the net degree change in body axis reached 360 degree in the horizontal plane between two time points when the monkey faced to the camera, the motion was scored as either a left/right rotation. The APO-induced rotation is only consistently seen when >90% of nigrostriatal DA neurons have been lost [14] and thus can be used as an indication of severe loss of DA neurons in the SNc.

#### Respiration rate, body weight, blood collection

Respiration rate was counted for 1 minute while the monkeys sat quietly in the monkey cage by an observer with a stop-watch. Body weights were measured in a monkey chair. The venous blood collection (2-3ml from the superficial veins of the limbs) was carried out under the mild anesthesia of veterinary ketamine (0.5–0.7 ml, 0.15 mg/ml, i.m.). An automated complete blood count was taken on a Sysmex XT2000i Automatic Hematology Analyzer (Lincolnshire, USA). The main indicators measured included red blood cell (RBC), white blood cell (WBC), platelet (PLT) and hemoglobin (HGB) counts. The blood chemistry panel was analyzed in a Roche Cobas c311 Automatic Biochemistry Analyzer (Basel, Switzerland). The main indicators measured included alanine aminotransferase (ALT), aminotransferase (AST), creatinine kinase (CK), and lactate dehydrogenase (LDH).

#### Brain tissue preservation

No control group monkeys were sacrificed for immunohistochemistry because it is well documented that intraparenchymal infusion of 10 μl saline causes no damage, both behaviorally and morphologically [15]. Although both DA and noradrenergic neurons in the brain are tyrosine hydroxylase positive (TH+), TH+ neurons in SNc and VTA are exclusively DA neurons [1,16]. Thus the brains from monkeys in the MPP^+^ group were prepared for TH+ cell counting under immunohistochemical staining in SNc and VTA. TH immunohistochemical staining of striatum was also performed to illustrate the loss of corresponding DA projections in striatum.

The monkeys were sacrificed under euthanasia and then were perfused by saline and paraformaldehyde after 28 weeks. For euthanasia, the monkeys were anesthetized with hydrochloric acidulated ketamine (0.3 g/2 ml, 0.5–0.7 ml, i.m., Jinlu, Shenyang, China) and then were given overdose pentobarbital (2–3 times than normal anesthesia). The brains were removed instantly from the skull and kept in formalin for pathological examination. The brains were then equilibrated in 20%-30% sucrose until they sank (usually in two weeks) to the bottom of the solution. Frozen sections (20 μm thick) were prepared on a Leica CM1850 UV (Solms, Germany) and were stored at -20°C. The sections, containing all of the SNc, VTA and striatum, were mounted on slides. For each monkey, each region (SN / VTA / striatum), and each side (left / right), five slides at the same interval (~1 mm for SN and VTA, ~4 mm for striatum) from rostral to caudal aspect of each region were counted.

#### Immunohistochemical staining of tyrosine hydroxylase

Briefly, 10% goat serum (Maixin, Fuzhou, China) was used as the serum blocker for 15 minutes at room temperature. Then anti-tyrosine hydroxylase primary antibody (AB152, Millipore, USA, diluted 1:1000 with 0.01M PBS) was applied at 4°C overnight. Slides were rinsed with 0.01 M PBS-0.1% Triton X-100 (Solarbio, Beijing, China) and were incubated in biotin conjugated secondary antibody (1:150, Maixin, Fuzhou, China) for 30 minutes, and then incubated in streptavidin conjugated HRP (1:150, Maixin, Fuzhou, China) for 15 minutes at room temperature. DAB (DAB-1031Kit 20×, Maixin, Fuzhou, China) was used as the chromogen. Then the slides were rinsed with TO (xylene substitute, Cengxi, Guangxi China), gradient ethanol and distilled water. Then they were stained in Cresyl Violet acetate (C5042-10G, Sigma, USA) plus anhydrous sodium acetate and acetic acid, namely Nissl stain, for 20 minutes at room temperature, followed by sequential rinsing in distilled water, gradient ethanol (70%, 80%, 95% alcohol, 1 minute each) and a differentiation solution (ethanol: chloroform: diethyl ether = 1:1:1) for 5 minutes. Finally, the slides were dehydrated with ethanol, cleared with TO and closed with neutral balsam.

### Statistical analysis

DA neuron counting was carried out under the microscope (OLYMPUS CX41, OLYMPUS DP25 camera, cellSens Entry 1.4.1 software, Japan) with a field of view size 690.8 μm × 518.1 μm (20x) in the center of the SNc or VTA. To remove the quantification bias, ImageJ (version 1.48) was used for automatic quantification of neurons with following code:

run("8-bit");

run("Enhance Contrast", "saturated = 0.35");

run("Threshold…");

waitForUser("Set the Threshold");

run("Analyze Particles…", "size = 2000–20000 circularity = 0.05–1.00 clear include summarize");

TH+ projection quantification was carried out under the microscope with a field of view size 349.4 μm × 259.0 μm (40x) in the center of the striatum (putamen). ImageJ was also used for automatic quantification of projection area with following code:

run("8-bit");

run("Enhance Contrast", "saturated = 0.35");

run("Threshold…");

waitForUser("Set the Threshold");

run("Analyze Particles…", "size = 400–20000 circularity = 0.00–1.00 show = Outlines display summarize in_situ");

All of the DA neuron counting results were converted into percentages (to right SNc) and analyzed with related-samples Wilcoxon signed rank test in SPSS (provided by Zhejiang University). The percentages of area of TH+ projections in the striatum were also analyzed with a related-samples Wilcoxon signed rank test.

Repeated measures analysis of variance (ANOVA) was used to analyze the Kurlan’s score, rotation, respiratory rate, weight, blood cell count and chemistry panel in the SPSS software (Table 2). If the sphericity assumption appeared to be violated (P value <0.05 or null), a Greenhouse-Geisser adjustment was used. The main effects of week, MPP^+^, week×MPP^+^, and APO×MPP^+^ (only for rotation) were analyzed and considered as the indications of time stability, MPP^+^ effect, trend difference between groups, and rotation response difference between groups, respectively. For tests which had more than one measure, multivariate and univariate results were both considered.

**Table 2:** Applications of the repeated measures ANOVA.

	Within-subject factor	Between-subject factor	Measures
**Kurlan’s score**	time (week)	group (MPP^+^/Saline)	Score
**Rotation**	time (week), APO (Y/N)	group (MPP^+^/Saline)	left rotation count, right rotation count
**Respiratory rate**	time (week)	group (MPP^+^/Saline)	rate (/min)
**Weight**	time (week)	group (MPP^+^/Saline)	weight (kg)
**Blood cell count**	time (week)	group (MPP^+^/Saline)	RBC, WBC, PLT, HGB
**Chemistry panel**	time (week)	group (MPP^+^/Saline)	ALT, AST, CK, LDH

The levels of significance were considered to be P < 0.05 and P < 0.01. Data was presented as the mean and standard deviation in all figures.

## Results

### Complete and specific lesion

**Fig 2:**
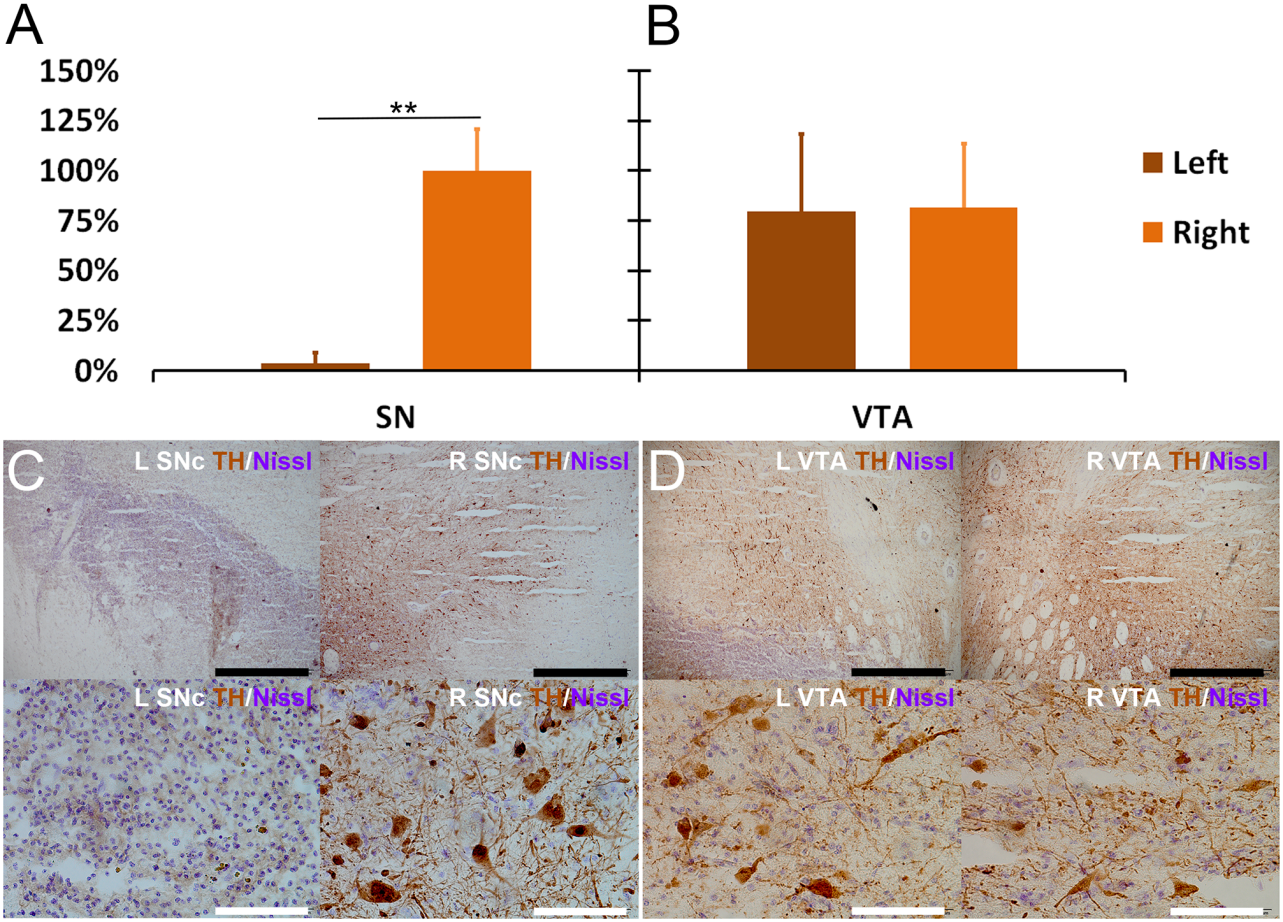
Complete and specific lesion induced by MPP^+^. Monkey #1 in the MPP^**+**^ group unexpectedly died 2 weeks after the infusion operation. The other three monkeys in the MPP^**+**^ group were sacrificed 28 weeks after the MPP^**+**^ infusion. The pattern and degree of the cellular loss in monkey #1 were quite similar to other three brains observed in the end of the experiment. Thus, four monkeys were under statistical analysis together. The MPP^**+**^ infused monkey had severe lateral depletion of DA neurons in the SNc, while preserving DA neurons in the VTA. **(A)** Nonparametric median test showed significant difference of TH+ neuron counting between left and right SNc (**P < 0.001). **(B)** Nonparametric median test showed no difference of TH+ neuron counting between both VTA (P = 0.904). **(C)** Microscopic images of the left and right SNc. No TH+ neurons were found in the left SNc; while normal TH+ neurons with proper staining, clear projections and intact soma were found in the right SNc. **(D)** Microscopic images of the left and right VTA. Normal TH+ neurons with clear morphology were found in both left and right VTA. Scale: white bar = 100 μm, black bar = 1000 μm.

**Fig 3:**
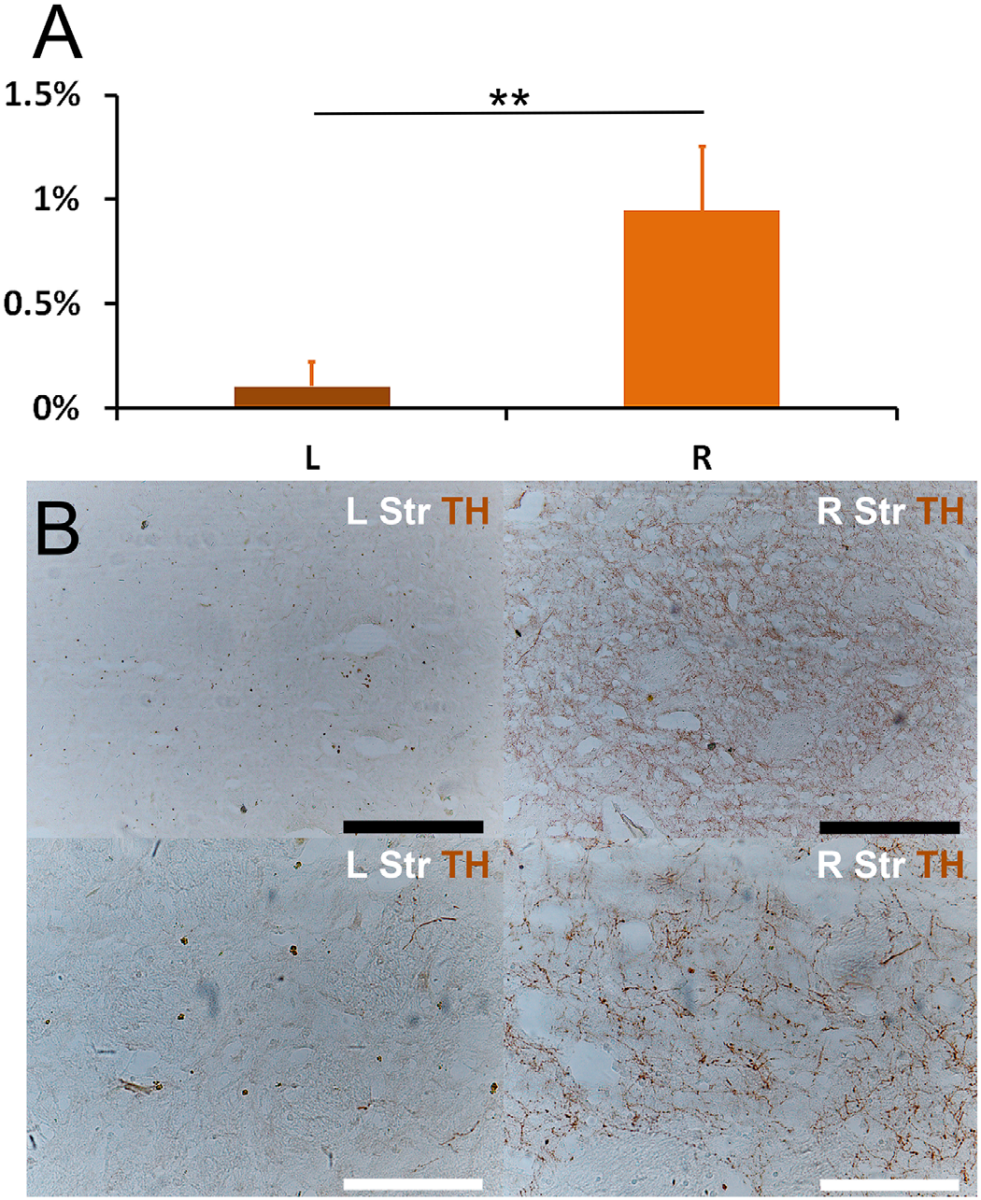
Loss of TH+ projections in striatum. The MPP^**+**^ infused monkey had severe lateral loss of TH+ projection. **(A)** The percentage of area of TH+ projection was defined as the ratio of TH+ area / total area in a particular field of view, and was automatically calculated with ImageJ. A nonparametric median test showed significant difference between left and right striatum (** P < 0.001). **(B)** Microscopic images of the left and right striatum. Right striatum had clear DA projections (TH+), which were stained as spots (transverse section) or fibers (longitudinal section). In contrast, left striatum had few stained spots and fibers, which suggested a remarkable loss of DA projections. Scale: white bar = 200 μm, black bar = 400 μm.

All of the brains in the MPP^+^ group were presented and used in final pathological examinations and statistical analysis. The SNc and VTA area were evaluated for tyrosine hydroxylase (TH) immunohistochemical staining. The absolute counting results of TH+ cells in the central area (690.8 μm × 518.1 μm) of SN and VTA for each monkey are shown in Table 3. To illustrate the percentage of DA neurons loss, the counting data of each monkey was converted into percentages (with the average counting of right SN set as 100% for each monkey) and were tested with a related-samples Wilcoxon signed rank test (Fig 2). The results of cell counting showed almost complete DA neuron loss (>95%, in terms of the average counting) in the left SNc (Fig 2A and 2C). There was also no TH staining difference between left and right ventral tegmental area (VTA) (Fig 2B and 2D). The results of TH staining of striatum showed that the TH+ projections in left striatum had a dramatically loss comparing with the right striatum (Fig 3). Monkey #1 in the experimental group unexpectedly died two weeks after the infusion operation from non-related lung complication. Surprisingly, its pattern and degree of the cellular loss were quite similar to other brains observed in the end of the experiment. Therefore, four monkeys were under statistical analysis together.

**Table 3:** TH cell counting for each monkey.

Monkey	SN		VTA	
	Left	Right	Left	Right
**#1**	1.0±1.5	52.2±7.0	39.0±3.9	42.0±8.1
**#6**	1.4±2.3	36.8±9.2	33.0±8.8	26.4±14.2
**#9**	2.6±2.2	39.2±27.6	28.2±11.5	35.4±18.0
**#10**	1.0±1.3	45.0±7.9	36.8±2.1	37.4±4.8

These absolute cell numbers were counted under an area of 690.8 μm × 518.1 μm in the central part of SN or VTA.

### Lasting rotation behavior

**Fig 4:**
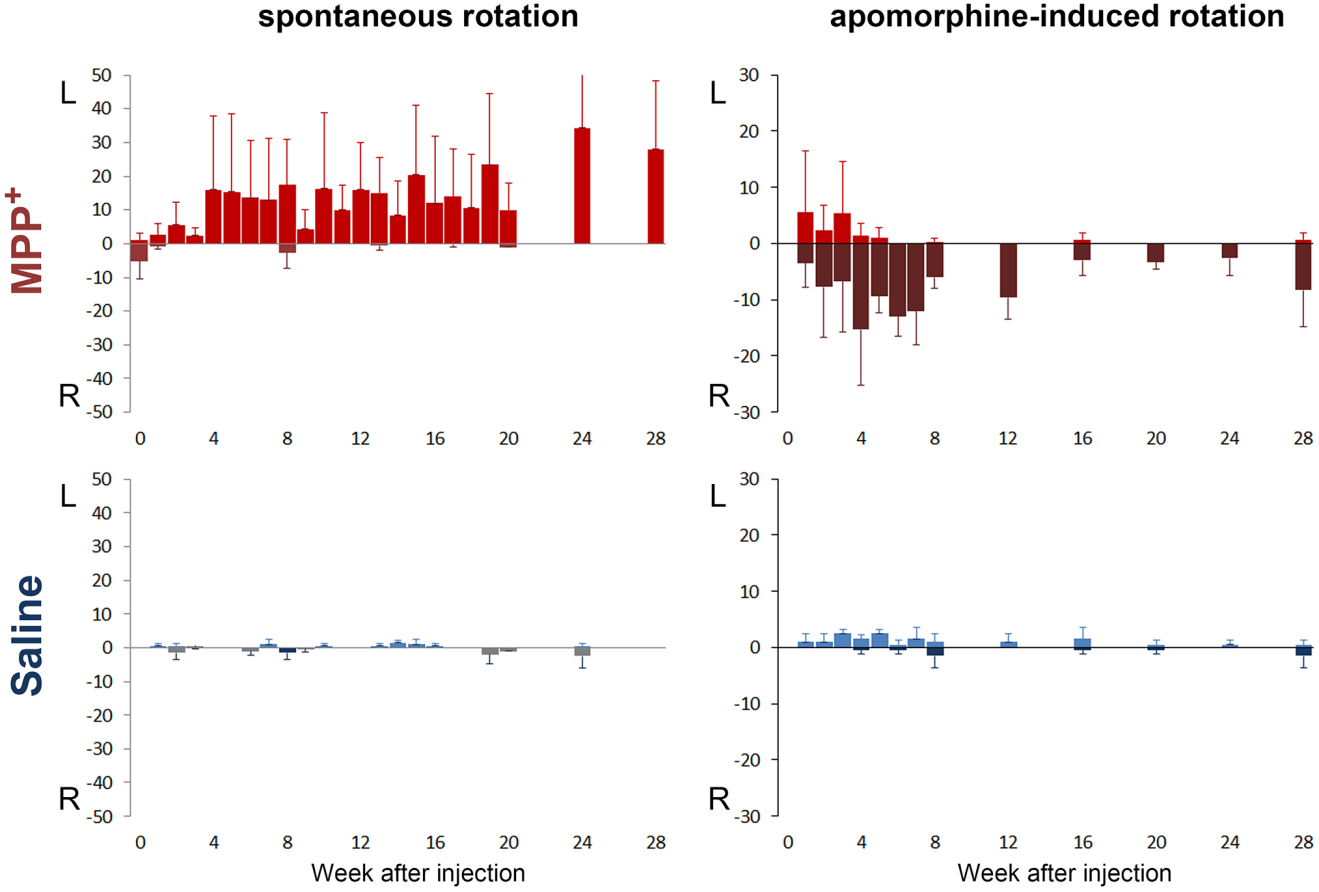
Lasting rotation behavior in the monkeys. Four panels show the spontaneous and APO-induced rotation counts in MPP^**+**^ (red, N = 3) and control (blue, N = 2) monkeys over 28 weeks. The ordinate represents the rotation direction (positive and lighter bars for left, negative and darker bars for right) and the number of rotations each week. Spontaneous rotations were counted during weeks 0–20, 24 and 28. APO-induced rotations were counted during weeks 1–8, 12, 16, 20, 24 and 28, respectively. The MPP^**+**^ monkeys developed a stable spontaneous left rotation behavior and were reversed to the right by APO, while control monkeys only displayed a few casual rotations throughout the 28 week period. There was a significant interaction effect of APO×MPP^**+**^ on APO-induced right rotation [Greenhouse-Geisser (GG), P = 0.039]. No significant effect of week was revealed (multivariate, Pillai’s Trace, P = 0.781; univariate, left, GG, P = 0.487; univariate, right, GG, P = 0.477) which indicated stable rotational behaviors for at least 28 weeks.

**Fig 5:**
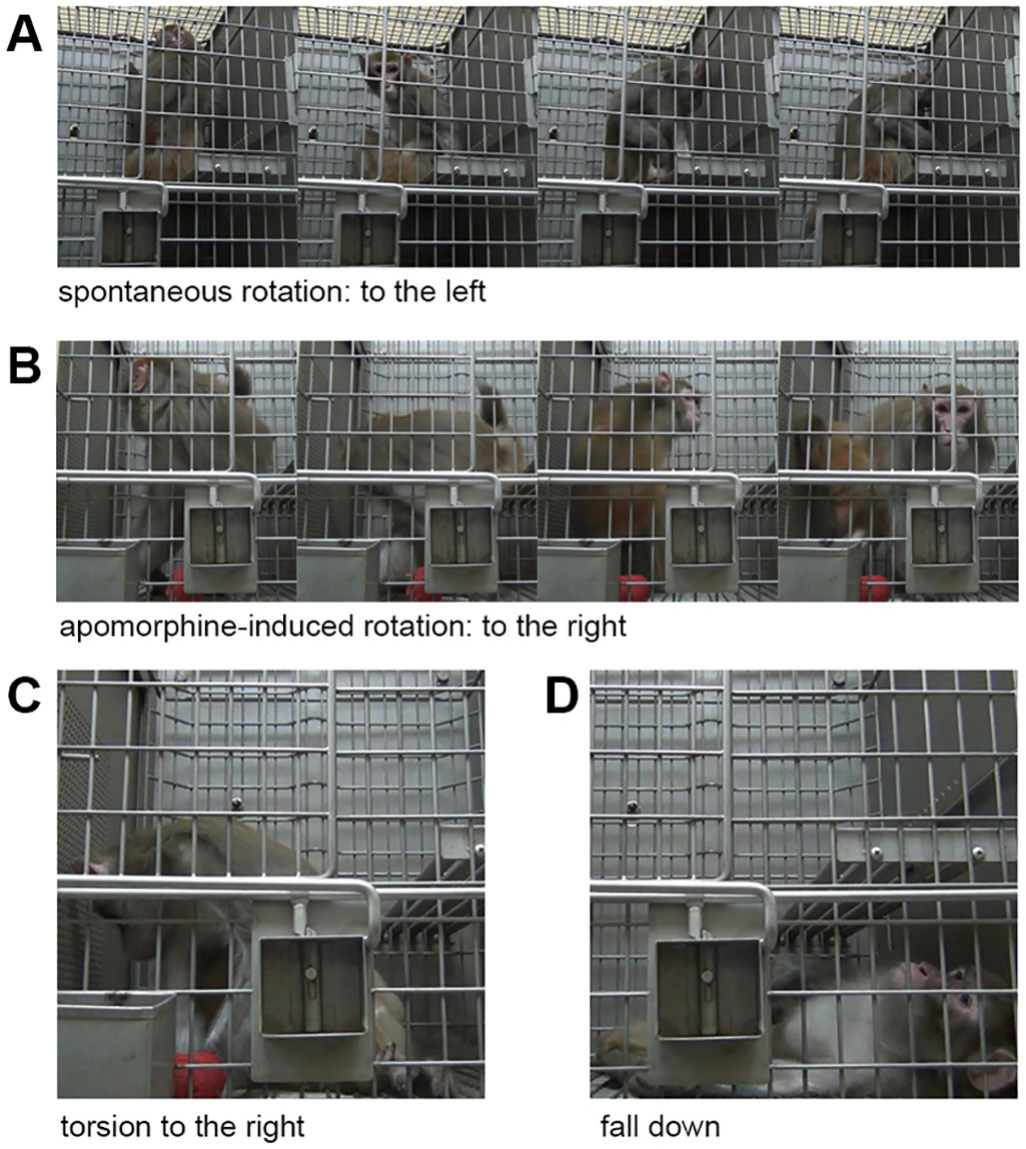
Representative images of the rotation and torsion of MPP^+^ treated monkeys. **(A)** A spontaneous rotation (to the left side of body) occurred when the monkeys were sitting without any disturbance. **(B)** An APO-induced rotation (to the right side of body) occurred 15 minutes after APO (1 mg/ml; 2 ml, i.m.) injection. **(C)** During the APO-induced rotation, the monkeys had a tendency to stop rotating and maintain a torsion posture for several to tens of seconds. The body was extremely distorted, with head turning right and backwards. **(D)** Sometimes a severe torsion might even lead to the loss of balance and falling down.

Four weeks after the infusion operation, the MPP^+^ infused monkeys developed unbalanced spontaneous rotation (Fig 4. upper left and Fig 5A.). Controls did not display this kind of spontaneous rotation (Fig 4. lower left). The spontaneous rotation occurred to the left side of body when the monkeys were sitting and walking without disturbance and increased when they were alert or frightened. In addition, after treated with APO, the MPP^+^ infused monkeys displayed intense rotation to the right side of body (Fig 4. upper right and Fig 5B), but the control monkeys did not display similar responses (Fig 4. lower right). The significant APO×MPP^+^ interaction effect (P = 0.039) indicated that the MPP^+^ infused monkeys had more APO-induced rotations than controls. As APO-induced rotation usually indicates a unilateral loss of at least 90% of DA neurons [14] and postsynaptic receptor supersensitivity [17], this neuropharmacological behavioral confirmed an unbalanced functional change of the nigrostriatal DA pathway. Moreover, statistical analysis indicated that this unbalanced rotation behavior lasted for at least 28 weeks during the observation.

It was also noticed that the APO induced an alternative behavior, body torsion, in the MPP^+^ treated monkeys in addition to the APO induced rotation. The body torsion occurred during rotation, usually in the middle of the rotating phase. When torsion began, the monkey stopped rotating, and stood or sat with whole body distorted (Fig 5C). Sometimes the torsion continued for tens of seconds until the monkey lost its balance and fell down (Fig 5D). This phenomenon may have influenced the total rotation count, but did not affect the lasting presence of the rotational behavior in the MPP^+^ monkeys

### Rapid onset of stable Parkinsonian symptoms

**Fig 6:**
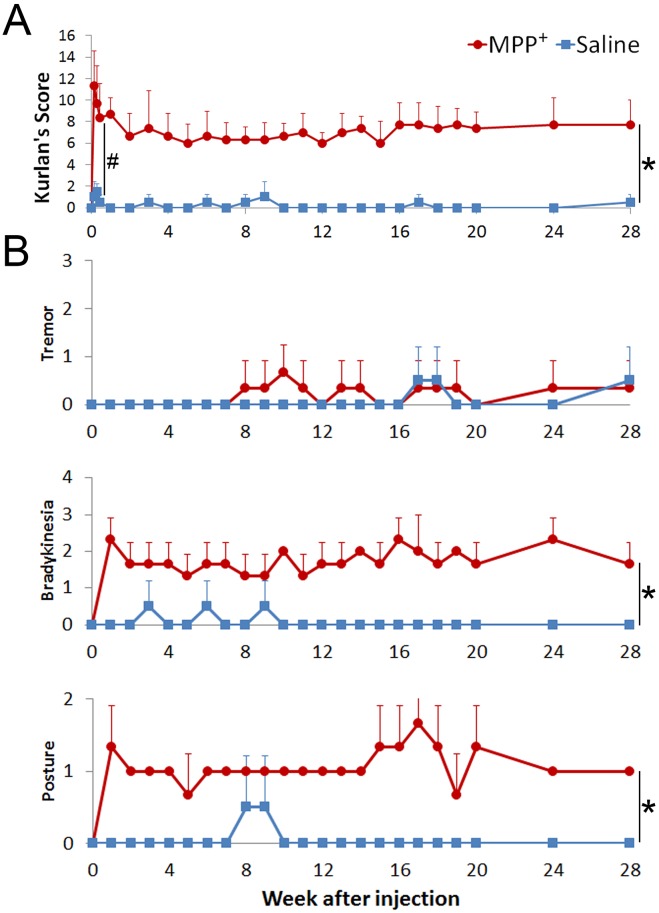
Quick-then-stable Parkinsonian symptoms. **(A)** Kurlan Scores of monkeys administered MPP^**+**^ (N = 3) or saline (N = 2) directly to the SNc and striatum. One day after the infusion operation, the Kurlan scores of the MPP^**+**^ infused monkeys rose markedly, followed by a partial fall into a stable plateau, while control monkeys recovered quickly (#. P = 0.032). The Kurlan scores of the MPP^**+**^ infused monkeys maintained a plateau for 28 weeks and were significantly higher than controls (main effect of group, *. P = 0.012). The scores of both MPP^**+**^ and control monkeys showed no changing trend (main effect of time, P = 0.434) and were parallel (interaction effect of time and group, P = 0.396). **(B)** Stable individual PD symptom scores. Specific items related to typical symptoms of PD in the Kurlan scale also showed long-term stabilization. The grading of bradykinesia and posture, but not tremor, had significant differences between MPP^**+**^ and control monkeys (*. P = 0.007 and P = 0.001, respectively).

One day after the infusion operation, control monkeys displayed a substantial recovery to normal status. MPP^+^ infused monkeys, on the other hand, displayed robust symptoms including stooped posture, abnormal gait, bradykinesia, unbalance, decline of gross motor skills, and defensive reaction, which were rated by Kurlan’s scale [12]. In general, the MPP^+^ infused monkeys had significantly higher Kurlan scores than control monkeys after the infusion (from day 0 to day 3; Fig 6A, #P = 0.032), which showed a rapid development of Parkinsonian symptoms.

Over the following seven months, the MPP^+^ infused monkeys had stable Kurlan scores of around 7, which were significantly higher (Fig 6A, *P = 0.012) than the controls, whose scores were around 0. The MPP^+^ infused monkeys also developed most of the core symptoms of PD, including bradykinesia, postural imbalance (Fig 6B.) and rigidity, with the exception of rest tremor, which was found to be intermittent and mild and not significantly different from the controls. Bradykinesia and posture imbalance, on the other hand, were very obvious in the MPP^+^ infused monkeys. In addition, distinct difficulty in passive movement (lead pipe rigidity) in right side limbs and axial torsion to the left were found in physical examinations. These symptoms were found to be stable for at least 28 weeks, which is much more stable than the classical MPTP method previously utilized [5]. The classical MPTP based approach usually requires 3–8 weeks of drug administration to reach a plateau (in contrast to one day witnessed here) and is followed by quick spontaneous recovery in several weeks after drug administration cessation [18].

### Good general condition

**Fig 7:**
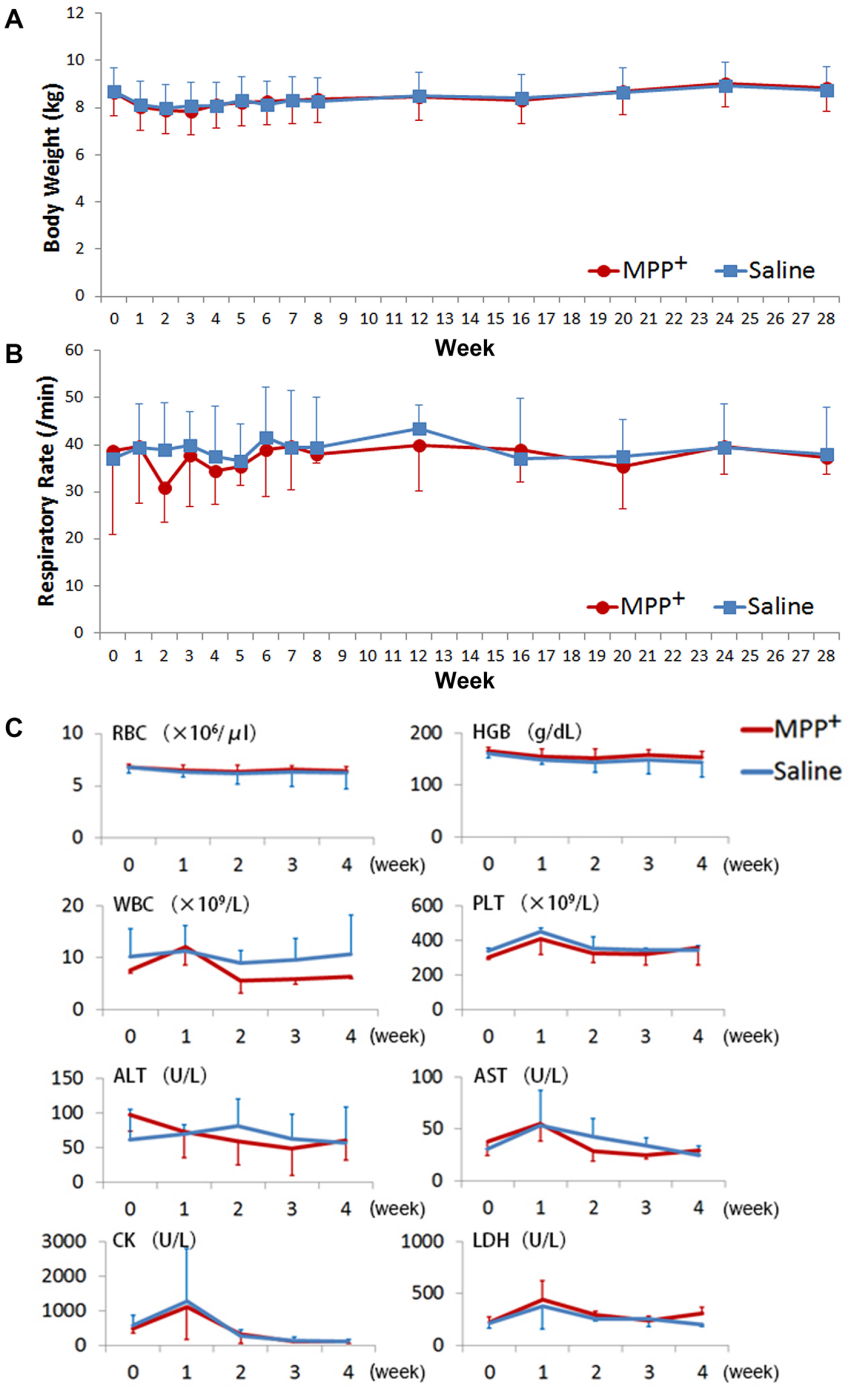
Body weight, respiratory rate, and venous blood test in the monkeys. No trend or significant differences of body weight and respiratory rate were revealed in and between MPP^**+**^ (N = 3) and control monkeys (N = 2) during the 28 week period. No trend difference between MPP^**+**^ (N = 3) and control monkeys (N = 2) were found in the blood samples [week×MPP^**+**^ interaction effect, P values for each count in parentheses: RBC (0.95), WBC (0.37), PLT (0.76), HGB (0.91), ALT (0.45), AST (0.51), CK (0.90), LDH (0.63); MPP^**+**^ main effect, P values for each count in parentheses: RBC (0.86), WBC (0.38), PLT (0.61), HGB (0.62), ALT (0.96), AST (0.75), CK (0.84), LDH (0.50)]. WBC, PLT, AST, CK, and LDH had a transient increase about a week after the operation, due to the operational damage to the surrounding tissues in both control and MPP^**+**^ infused monkeys.

Body weight and respiratory rate were monitored in this study to evaluate the general health status of the monkeys. Neither the MPP^+^ infused nor control group displayed a trend of increased or declined body weight (Fig 7A) or respiratory rate (Fig 7B) during the 28 week period [weight: week P = 0.088, week×group P = 0.788, group P = 0.993; respiratory rate: week P = 0.607, week×group P = 0.768, group P = 0.811]. No significant differences between MPP^+^ infused and control monkeys were found during the first month after the infusion operation in regards to complete blood counts (CBC) and blood chemistry levels (Fig 7C.). In both two groups, white blood cell (WBC), platelet (PLT), aminotransferase (AST), creatine kinase (CK), and lactate dehydrogenase (LDH) counts showed a transient increase one week after the infusion operation, but then returned quickly to baseline levels. This indicated a limited effect of the operation. No item implied the presence of a severe side effect caused by the operation or the intracerebral MPP^+^ infusion, which further suggested a good general health status in the monkeys with little to no non-specific effects on other systems.

## Discussion

### Rationale and advantages of route design

There are several DA systems in the body. This limits the efficacy of systematical DA neurotoxin infusions because they do not cause selective damage to one particular system, even when implemented in varying dosages (ranged from days to weeks) or route (via carotid artery, muscle, skin, or vein) regimes [4–6,19]. For example, systematical MPTP administrations, often considered as the “gold standard” of PD modeling, impair the digestive system [4,6] causing anorexia, weight loss, dehydration, somnolence, and even death [5]. In consideration of these non-specific dopaminergic damages, these lesioning methods are far from ideal for research aimed at the function of a specific dopaminergic pathway. To improve the efficiency and specificity of DA neurotoxin infusions for use in directed anatomical and pathological studies, such as those associated with DA neuron loss like Parkinson’s disease, this study developed a new experimental approach directed at lesioning the nigrostriatal DA pathway with MPP^+^, the toxic end-product of MPTP metabolism.

The paired SNcs are flat shaped and located in the narrow mesencephalon (Fig 1A), with the corticopontine and pyramidal tract on the ventral side, and the red nucleus, medial lemniscus (ML), medial longitudinal fasciculus (MLF), and mesencephalic reticular formation on the dorsal side. These structures make any infusion route passing through to the SNc difficult to negotiate without causing unnecessary damage to the surrounding structures. Therefore, the designed route utilized here featured an oblique approach to the SNc while passing through the striatum to provide a better solution in directly targeting the SNc. This route had the following advantages:

1. The route avoided passing through the related structures around the SNc, and thus reduced the risks of hemiplegia caused by damaging the pyramidal tract, of bradykinesia from damage to the red nucleus, or impaired balance and posture from operational damage to a sensory pathway (such as the ML and MLF). This route improved the specificity and safety of the surgery.
2. The route facilitated the delivery of neurotoxin into both the SNc and striatum at the same time. This dual intoxication enabled specific MPP^+^ uptake via DATs to occur on the surface of somas in the SNc and axons in the remote striatum [20]. As DA neurons located in the same region of the SNc have differing projections to other brain regions [1], the dual intoxication ensured that specific projections to the striatum received more MPP^+^ neurotoxin in comparison to other non-specific neurons, leading to more prominent DA neuron loss. Furthermore, the dual intoxication provided additional selectivity among adjacent DA related systems, such as the dopaminergic, catecholaminergic or serotoninergic system in the VTA, the posterior hypothalamus, the arcuate nucleus or the periventricular nucleus. All of these structures have been shown to be impaired with the systematical administration of MPTP [21–23]. Moreover, the dual intoxication method may limit any compensatory effect from projections of the contralateral SNc [24], which has been shown to cause up-regulation of the TH and DAT expression in the striatum when lesioning the SNc [25].
3. This route penetrating through the SNc provided the opportunity to inject neurotoxin into multiple sites of the SNc, which improved the ablation of DA neurons and reduced any compensatory mechanism of the remaining DA neurons [26,27].

### Rationale and advantages of toxin choice

The choice of MPP^+^ as the DA neurotoxin was based on following considerations. Previous studies have shown the toxic effect of MPTP to be dependent on the effects of its metabolite, MPP^+^, released from glial cells. MPP^+^ is directly and specifically absorbed into DA neurons by DAT [28] where it disturbs the mitochondrial respiratory chain [29], leading to the redistribution of DA and oxidative stress [30], and eventually causes DA neuron death [29,30]. The use of MPP^+^ instead of MPTP provides the following theoretical advantages:

1. It bypasses the metabolism of glial cells [31] and enhances the direct toxic effect (leading to complete ablation and stability of PD symptoms) as well as reduces the interference of medial factors (such as monoamine oxidase, MAO, and so on), which minimizes the individual variation in the monkeys.
2. The intracerebral administration of MPP^+^ is a necessity as the agent cannot pass through the brain blood barrier [21]. This route, therefore, fortifies the specificity of the agent to the brain and protects organs outside the CNS from the toxic effects of MPTP [4,6], which improves the general status of the animals.
3. MPP^+^ also has a number of obvious advantages in comparison to another traditional dopaminergic drug toxin, 6-hydroxydopa (6-OHDA). MPP^+^ has been found to evoke a more similar damage pattern witnessed in PD patients [32] and to produce lasting PD symptoms, as demonstrated here, while 6-OHDA does not produce permanent damage in rats and monkeys. For example, infusions of 6-OHDA into the nigrostriatal DA pathway of rats failed to produced lasting effects [33] and the intracerebral administration of 6-OHDA to rhesus monkeys only generated temporary and non-specific damage to dopaminergic and noradrenergic pathways with recovery in 3–4 weeks [34]. Moreover, MPP^+^ can be administered alone, but 6-OHDA needs to be administered with a norepinephrine reuptake inhibitor [14,35] to reduce non-specific toxication. In addition, MPP^+^ is easy to store and prepare, while 6-OHDA usually requires dark storage, an antioxidant agent and preparation immediately prior to use.

### Satisfied ablation and accessible technique

A “standard stereotaxic procedure” with MRI-compatible external markers developed previously in our lab provided accurate locations within 1 mm [36] in this study, which ensured the specific ablation of DA neurons. This method utilized external glycerol markers, instead of a plastic MRI-compatible frame, to avoid image distortions and reduce the size of the scanning coil needed [36]. This provided higher quality MR images. All told, this method is more convenient and economically feasible then conventional monkey MRIs which are dependent on an expensive MRI-compatible frame to orientate the monkeys. Our results confirmed the specificity and completeness of administering MPP^+^ directly to the SNc and striatum to destroy DA neurons, and was similar to the damage pattern in PD patients [37]. Also, behavioral data of the monkeys, following just one infusion of MPP^+^, confirmed stable Parkinsonian symptoms (for at least 28 weeks). Thus, this model may provide a very suitable platform for long-term experiments for potential PD therapies (such as stem cell transplantation), as the model provides stable symptoms without the toxic effects of subsequent and repeated MPTP/MPP^+^ administration. In addition, this method of ablating a targeted DA pathway developed here is applicable to a number of studies on DA pathway functions (e.g. the VTA in relation to reward or addiction). Moreover, the MRI stereotaxic targeting and direct lesioning technologies can be modified to investigate other neural pathway studies by utilizing the correct neurotoxin.

### Scarcity of rest tremor suggests different origin

In this study, rest tremor was found to be slight and only intermittently present after two months. Although this is consistent with previous modeling studies, which have seldom found that rest tremor is reproduced in PD monkeys [14,19,38,39], some reports suggest that MPTP-induced tremor occurs mainly on long-term and severe PD monkeys [18,40]. However, the behavioral phenotypes found in this study (with nearly complete lesioning in SNc, which was as severe as those monkeys with rest tremor in previous reports) provided direct evidence that the mere loss of nigrostriatal pathway (even with >95% DA neuron loss), at least within several months in rhesus macaque, is poorly correlated with rest tremor. This finding not only supports the idea that rest tremor in PD involves multiple regions, including the primary and supplementary motor cortex, sensory-parietal zones, thalamus, globus pallidus, and cerebellum [41–45], and suggests that the mere loss of the nigrostriatal pathway might not be sufficient to induce the needed changes in these regions to cause rest tremor. This implies that the rest tremor seen in other reports may be due to a complex interaction of multiple (DA or other) systems.

Interestingly, the treatment of animal models with different drugs has shown diverse effect on typical PD symptoms. For example, our previous study utilizing MPTP induced monkeys found four typical PD symptoms (tremor, bradykinesia, imbalance and defensive reaction) to be differentially affected by either morphine or l-3,4-dihydroxyphenylalanine (L-Dopa) [40]. L-Dopa displayed a therapeutical effect on bradykinesia but not on rest tremor, while morphine displayed significant improvements in rest tremor. This distinction also suggested that rest tremor involves more than the DA system alone.

Taken together, this study supports the notion that rest tremor has an origin different than a pure deficiency in nigrostriatal dopamine and is likely the outcome of long-term interactions between the basal ganglia loop and the cerebello-thalamo-cortical circuit [39]. As such, this study on the complete ablation of nigrostriatal DA neurons also supports the view that PD has a pathophysiology that involves the failures of multiple neurotransmitter systems or neural circuits, in addition to dopamine or the basal ganglia. It also suggests that the continuing search for potential non-dopaminergic treatments is reasonable in PD research.

### Limitations

This study was a pilot exploration. Although there were significant differences between MPP^+^ and control monkeys with high statistical power, the small group sizes were used to evaluate the surgery and toxin administration. In the future, increased sample sizes and more practical application of this lesioning method and PD model will provide information to further improve the approach as well as a better validation of its use in clinical development. As PD is an age-related neurodegenerative disease, aged monkeys should be considered in future modeling.

It is also apparent that this acute hemi-Parkinsonian model may be good at imitating only the initial step (asymmetrical onset of symptoms) or the final step (nearly complete loss of DA neurons) of PD patients but not the whole progressive course of the disease. Thus, it requires a more cross-sectional view of its usefulness, rather than a longitudinal one. Indeed, the chronic process of DA neural loss is important to understanding the pathogenesis of PD. However, the usefulness of this acute model to attain PD-like monkey models in short time frames provides a stable foundation for investigating novel treatments that can target both the initial or late stages of the disease. In addition, this model can be used supplementary in longitudinal studies developed in chronic transgenic models to elucidate specific pathway determinants.

### Implications for DA pathway research

This study not only established a method for specifically and completely ablating a certain DA pathway, but also, as an exemplary embodiment, provided more specific information about the nigrostriatal DA pathway. All-told, this acute-MPP^+^ infusion model better fits the criterion of an ideal animal model for studying DA pathways [46] because it had a demonstrated loss of DA neurons, detectable symptoms, and a relatively short preclinical course (which is economically and experimentally efficient). This kind of hemi-Parkinsonian monkey is also useful for exploring novel antiparkinsonian therapies, including surgical interventions. [46] It also largely improved animal welfare in the studies, which will, without a doubt, improve the overall quality of science in long-term studies. This approach it is a promising prospect for future investigations into addiction, Parkinson’s disease and other DA related physiological or pathophysiological topics, and will have an important role in providing new translational knowledge for clinical treatment.
