# Correction: 1-Methyl-4-Phenylpyridinium Stereotactic Infusion Completely and Specifically Ablated the Nigrostriatal Dopaminergic Pathway in Rhesus Macaque

**DOI:** 10.1371/journal.pone.0147094

**Published:** 2016-01-08

**Authors:** Xiaoguang Lei, Hao Li, Baihui Huang, Joshua Rizak, Ling Li, Liqi Xu, Li Liu, Jing Wu, Longbao Lü, Zhengbo Wang, Yingzhou Hu, Weidong Le, Xingli Deng, Jiali Li, Yonggang Yao, Lin Xu, Xintian Hu, Baorong Zhang

Dr. Tianzhuang Huang is not included in the author byline. He should be listed as the seventeenth author and affiliated with Yunnan Key Laboratory of Primate Biomedical Research, Kunming, Yunnan, China. The contributions of this author are as follows: Contributed reagents/materials/analysis tools and performed the experiments.

There are errors in the Funding section. The correct funding information is as follows: This work was supported by National Program on Key Basic Research Project (973 Programs 2015CB755605, 2012CB825503, 2012CBA01304), the National High-Tech R & D Program of China (863 Program 2012AA020701), the Strategic Priority Research Program of the Chinese Academy of Sciences, Grant No. XDB02020005, the Key Research Program of the Chinese Academy of Sciences, Key Program of the Chinese Academy of Sciences (KZCC-EW-103-2), Training Program of the Major Research Plan of the National Natural Science Foundation of China (91332120), National Natural Science Foundation of China (81471312, 31271167, 81271495, 81271248, 81200984), the Yunnan Provincial Project to Attract One-hundred Exceptional Talents from Overseas, and Selected Frontier Scientific Significant Breakthrough Project of CAS, International Cooperation Projection (2011C14026) of Zhejiang Province Department of Science and Technology. The funders had no role in study design, data collection and analysis, decision to publish, or preparation of the manuscript.
